# Understanding the effects of intramuscular injection and feed withdrawal on *Salmonella* Typhimurium shedding and gut microbiota in pullets

**DOI:** 10.1186/s40104-021-00597-9

**Published:** 2021-06-04

**Authors:** Nitish Narendra Joat, Samiullah Khan, Kapil Chousalkar

**Affiliations:** School of Animal and Veterinary Sciences, The University of Adelaide, Roseworthy, South Australia 5371 Australia

**Keywords:** Corticosterone injection, Feed withdrawal stress, Gut microbiota composition, Layer chicken, *Salmonella* Typhimurium infection

## Abstract

**Background:**

Gut microbiota plays a key role in health, immunity, digestion, and production in layers. Factors such as environment, diet, diseases, stress, and flock management significantly affect gut microbiota; however, it is not known how potential stressors such as intramuscular injections or feed withdrawal alter the composition of gut microbiota that result in increased the shedding level of foodborne pathogens. In the current study, the effects of intramuscular corticosterone injection and feed withdrawal were evaluated to understand their role in *Salmonella* Typhimurium shedding and changes in the composition of gut microbiota in layers.

**Results:**

*Salmonella* shedding was observed for 8 weeks post-infection. There was a significant increase in *Salmonella* Typhimurium count after intramuscular injection and feed withdrawal. The *Salmonella* infected and the negative control groups showed significant differences in the abundance of different genera in gut microbiota at week 1 and up to week 7 post infection. The infected group showed a significant reduction in alpha diversity of gut microbiota. Firmicutes reduced significantly (*P* < 0.05) after intramuscular injection, while the feed withdrawal groups did not cause any significant changes in Proteobacteria-Firmicutes ratio. Furthermore, intramuscular injection resulted in a significant change in alpha diversity of gut microbiota.

**Conclusions:**

Exposure of chicks to relatively low dose of *Salmonella* Typhimurium can lead to persistent shedding in pullets. The *Salmonella* Typhimurium infection disrupted the gut microbiota composition immediately after infection. The potential stress of intramuscular injection and feed withdrawal significantly increased the *Salmonella* Typhimurium count in faeces. The intramuscular injection also resulted in a significant alteration of the Proteobacteria-Firmicutes ratio, which could increase the risk of dysbiosis.

**Supplementary Information:**

The online version contains supplementary material available at 10.1186/s40104-021-00597-9.

## Introduction

The gut microbiota is a subject of investigation owing to its key role in homeostasis, nutrient digestion, immune modulation and conferring protection against pathogen colonisation in host [1]. In the last two decades, significant advances have been made in gut microbiota research due to tools such as 16S rRNA and whole genome sequencing [2]. Because of the diverse functions of gut microbiota especially its role in the immune system modulation, nutrient digestion, and colonisation resistance to pathogens, it is essential to understand its role in food-producing animals such as layer chickens.

Layer farming is one of the leading food-producing sectors in the world because of the acceptability and high demand for egg and egg products. Foodborne pathogens such as *Salmonella* and *Campylobacter* are global concerns for the egg industry. *Salmonella enterica* is commonly related to contaminated eggs and egg products that act as a source of infection in humans. Globally, *Salmonella* Enteritidis is the dominant serotype [3]; however, in Australia, *Salmonella* Typhimurium is the dominant serotype, which has been responsible for multiple egg and egg product related outbreaks in humans [4]. *Salmonella* Typhimurium infection in adult hens does not induce clinical signs; therefore, the hens act as a carrier by continuously shedding it to the environment [5]. It has been reported that *Salmonella* shedding in hens is intermittent and the bacterium can remain undetected for several weeks [5]. In porcine alveolar macrophages and in mice, repeated subcutaneous dexamethasone injections have been shown to increase the shedding of *Salmonella* Typhimurium [6].

The impact of *Salmonella* Enteritidis and *Salmonella* Typhimurium colonisation on the diversity of gut microbiota in chicks has been reported earlier [7, 8]. However, the *Salmonella* Enteritidis challenge study was conducted over a short period and the *Salmonella* Typhimurium study was conducted in adult hens. The life cycle of commercial layer birds is up to 80 weeks that involves rearing for up to 16 weeks. The gut microbiota acquisition, establishment and development start from the early age [9]. Thus, it is essential to understand how the colonisation of *Salmonella* Typhimurium during rear affects the gut microbiota composition. Furthermore, pullets go through multiple potential unavoidable stressors during rearing that include vaccinations (intramuscular or sub cut injections), transport to production shed and on-set of lay. These stressors activate the hypothalamic-pituitary-adrenal axis (HPA axis) in the host [10]. This triggers the neuroendocrine pathway that leads to the secretion of stress hormones (glucocorticoids and catecholamines) [10–12]. Out of these hormones, corticosterone (glucocorticoid) is the main stress hormone in birds that affects the gut microbiota and results in the imbalance of beneficial (Firmicutes) and pathogenic (*Mycoplasma*, *Campylobacter*, *Pseudomonas* etc) microbial taxa in wild birds [13]. An earlier study in broilers also reported that acute stressors can affect the normal intestinal microbiota and intestinal structure, which could lead to increased *Salmonella* Enteritidis colonisation [14]. Based on relevant studies described earlier, it is evident that stressors in adult hens can lead to the replication of *Salmonella* Typhimurium and can negatively impact the gut microbiota. The effects of stress on gut microbiota and *Salmonella* Typhimurium shedding have been documented in literature [13, 15]. Furthermore, the effects of *Salmonella* Typhimurium colonisation on gut microbiota in adult hens are also reported [8]. However, there is a scarcity of data on the interaction between gut microbiota and *Salmonella* Typhimurium during stressful events. Therefore, the objective of this study was to investigate the effects of *Salmonella* Typhimurium challenge on the gut microbiota of pullets. This study also investigated the effects of feed withdrawal and intramuscular injection on gut microbiota composition and *Salmonella* Typhimurium shedding pattern in pullets.

## Methods

### Animal ethics committee approval

The animal trial was approved by the Animal Ethics Committee at The University of Adelaide under approval number: S-2019-004.

### Hatching and rearing of *Salmonella* free laying chicks

Fertile eggs of an Isa-Brown parent flock were obtained from a commercial hatchery. The eggs were fumigated and incubated for 21 d at 37.5 °C. Relative humidity in the incubator was 55% until d 18 and was increased to 60% until d 21. A total of 76 chicks were raised in different pens during the experiment. Minimum number of birds in each group was 11. Earlier studies included 3 [16–19] and 7 [8] birds per group to understand the effect of *Salmonella* Typhimurium infection on gut microbiota composition in chickens. The chicks were housed in separate rooms at the Roseworthy Campus, The University of Adelaide. The housing conditions (size of pens, stocking density, lighting program etc) were according to Australian animal welfare standards and guidelines [20]. The birds were reared on mesh floor and had some access to faeces. Swabs were collected from the incubator after hatch and processed for *Salmonella* isolation. Before the placement of chicks, pens and facility were cleaned and decontaminated using Saniguard (Chemetall, Australia) followed by formaldehyde fumigation. The chicks were provided with ad libitum feed and water throughout the experiment [21]. The chicks were divided into six different treatment groups as outlined in Table 1.

**Table 1:** Details of various treatment groups used in the study

Sr. No.	Treatment Code	Treatment group	No. of chicken
1	NC_CORT	Negative control + CORT	11
2	INF_CORT	*Salmonella* Typhimurium+ CORT	14
3	INF_PBS	*Salmonella* Typhimurium + PBS	13
4	NC_FW	Negative control + Feed Withdrawal	12
5	PC	*Salmonella* Typhimurium	14
6	INF_FW	*Salmonella* Typhimurium + Feed withdrawal	12

*CORT* Corticosterone, *PBS* Phosphate buffered saline, *NC* Negative control, *PC Salmonella* Typhimurium challenged, *FW* Feed withdrawl

### Bacterial strains and challenge

Bacterial strain KC 30 (*Salmonella* Typhimurium phage type 9) was cultured on nutrient agar overnight at 37 °C. This strain was isolated from an egg farm during a previous epidemiological investigation [5]. Twenty-four hours before infecting the chicks, a single colony was incubated in 10 mL Luria Bertani (LB) broth for 6 h in a shaking incubator (180 r/min). From this culture, 10 μL was subcultured in 30 mL LB broth at 37 °C overnight. On the day of chicks challenge, the broth was centrifuged, and bacterial pellet was washed with phosphate buffered saline (PBS). The pellet was resuspended in 25 mL PBS and the optical density of the suspension was measured at 600 nm.

In the challenged groups, individual chicks received 10^3^ colony forming units (CFUs) of *Salmonella* Typhimurium orally at d 7 of chicks’ age (treatment groups- INF_CORT, INF-PBS, PC and INF_FW), while the control groups were sham inoculated with sterile PBS (treatment groups – NC_CORT and NC_FW). The dose rate was kept low to understand the effect of *Salmonella* Typhimurium colonisation on gut microbiota [16, 17]. The chicks were monitored for clinical signs of salmonellosis for 8 weeks. The leftover inoculum was maintained on ice, serially diluted and plated onto Xylose Lysin Deoxycholate agar (XLD) to confirm the exact dose received by the individual chicks. Faecal samples were collected every week from the individual chicks to monitor the *Salmonella* Typhimurium shedding. A subset of faecal samples was stored at − 20 °C for DNA extraction and 16S rRNA sequencing.

### Intramuscular injection and feed-withdrawal at week 7 of chicken age

An injectable solution was prepared by dissolving the corticosterone-HBC complex (Merck, Australia) in water to make the final dose as 1.5 mg/kg body weight. At week 7 post-infection, chickens in treatment groups negative control corticosterone (NC_CORT) and *Salmonella* Typhimurium corticosterone (INF_CORT) received an intramuscular injection of corticosterone, while group *Salmonella* Typhimurium PBS (INF_PBS) was injected PBS. The week 7 post-infection time point was selected to mimic field conditions to understand the effects of potential stress caused by intramuscular injection. The common intramuscular vaccines administered in layer chickens in the field are fowl cholera, egg drop syndrome, and Newcastle disease [22]. In this study, birds did not receive any vaccine. Group negative control feed withdrawal (NC_FW) and group *Salmonella* Typhimurium feed withdrawal (INF_FW) were subjected to feed withdrawal for 4 h immediately after night photoperiod. It has been noted that commercial layers are likely to go through feed withdrawal stress at some point in 80 weeks of lifespan [23]. The feed withdrawal groups were included to mimic the field conditions. The group positive control (PC) was not subjected to any physical or hormonal treatment. From all the treatment groups, faecal samples were collected 24 h after the injection or feed withdrawal for bacterial enumeration and the chickens were humanely sacrificed at 72 h following the injection or feed withdrawal. At the time of sacrifice, cloacal swabs and tissue samples from liver, spleen, ileum, caecum and colon were collected from all the chickens for quantification of *Salmonella* Typhimurium through bacterial culture method.

### *Salmonella* detection in faeces and swabs

All the groups (positive and negative) were tested weekly for *Salmonella* Typhimurium according to a previously published protocol [5]. Briefly, faecal samples from pens or swabs collected from the hatching incubator were incubated in buffered peptone water (BPW) (Oxoid, Australia) at 37 °C overnight and 100 μL of it was transferred to Rappaport- Vassiliadis soya peptone (RVS) broth (Oxoid, Australia). The RVS samples were incubated at 42 °C for 24 h and streaked on XLD plates and incubated overnight at 37 °C. The *Salmonella* suspected colonies from the XLD were subcultured onto Brilliance *Salmonella* agar (BSA) plates (Oxoid, Australia).

### *Salmonella* Typhimurium enumeration in tissues

Ileum, caecum, colon, liver and spleen were collected at 8-week post-infection for the enumeration of *Salmonella* Typhimurium. Briefly, 0.1 to 0.2 g of tissue was collected in sterile 1.5 mL microcentrifuge tubes containing stainless steel beads (0.5–2 mm) and PBS. The tissue samples were homogenised using bullet blender (Next Advance, USA) on full speed for 5–10 min. Serial ten-fold dilutions were prepared in PBS and 100 μL of the homogenates was spread plated on XLD media and incubated overnight at 37 °C. *Salmonella* load in tissue was expressed as mean log_10_ CFU/g of tissue. The negative samples from the *Salmonella* Typhimurium challenged groups after direct plating were enriched in BPW and RVS as described [5]. The samples that showed positive for *Salmonella* after enrichment were noted as “1” and the negative samples were noted as “0” for calculating the proportion of positive samples.

### DNA extraction from faeces

DNA was extracted and purified from faecal samples (*n* = 584) using the QIAamp Fast DNA Stool Mini kit (Qiagen, Australia) with a modified protocol [24] that was further optimised for this study. Briefly, 200 mg faecal samples were vortexed after adding 1 mL of preheated (70 °C) InhibitEx buffer. The samples were homogenised after the addition of glass beads (acid washed, 450–600 μm (180 mg) and 106 μm (210 mg)), using a bullet blender for 5 min. The samples were heated at 95 °C for 7 min and then incubated on ice for 30 s. The samples were centrifuged for 2 min and the supernatant was collected and further processed for DNA extraction according to the modified QIAamp Fast DNA Stool Mini kit protocol [24]. DNA was eluted in 100 μL of ATE buffer and stored at − 20 °C until used for qPCR and 16S rRNA sequencing.

### Quantitative PCR and optimization of standard curve for *Salmonella* Typhimurium

The extracted DNA samples were subjected to quantitative PCR for *Salmonella* Typhimurium quantification from week 1 post infection until sacrifice. The qPCR was performed using SensiFAST SYBER HI-ROX Kit (Bioline, Australia) following the manufacture’s protocol. The qPCR reaction was performed in 72 well rotor-gene disc (Qiagen, Australia). The master mix was prepared and added to the disc through Corbett CAS1200 robot (Corbett Life Science, Australia). The 10 μL final reaction volume contained 5 μL SensiFAST SYBER Hi-Rox mix, 1 μL each of the forward (5′-TTTACCTCAATGGCGGAACC) and reverse (5′-CCCAAAAGCTGGGTTAGCAA) TSR3 primers, 1 μL RNase-free water and 2 μL DNA template. The cycling conditions were initial denaturation at 95 °C for 3 min, then 40 cycles of denaturation at 95 °C for 5 s, annealing and extension at 59 °C and 72 °C for 20 and 30 s, respectively.

A standard curve was constructed by spiking non-infected control faeces with known amount of *Salmonella* Typhimurium. Briefly, serial ten-fold dilutions of *Salmonella* Typhimurium grown overnight in LB broth were used to spike the faecal samples. The DNA was extracted from the spiked faecal samples to construct a standard curve and limit of detection was established.

### 16S rRNA library preparation and illumina sequencing

Out of 584 extracted DNA samples, 360 DNA samples were submitted to the Ramaciotti Centre for Genomics (University of New South Wales, Australia) for 16S rRNA sequencing and generation of operational taxonomic units (OTUs) table using barcoding PCR. The total reaction volume was 25 μL containing 12.5 μL KAPA HiFi HotStart Readymix (Kapa Biosystems), 1 μL of each of the forward and reverse primers, 1 μL DNA sample and 10.5 μL PCR grade water. This PCR was used to generate 2 × 300 bp pair-end reads in Illumina. To generate the reads, V3-V4 region specific primer pair (341F 5′-CCTACGGGNGGCWGCAG-3′; 805R 5′-GACTACHVGGGTATCTAATCC-3′) was used. The thermal cycling conditions for the PCR were as follows: initial denaturation 95 °C for 3 min, followed by 35 cycles of denaturation at 95 °C for 30 s, annealing at 55 °C for 30 s and elongation at 72 °C for 30 s, ending with final elongation at 72 °C for 5 min. PCR products were normalised and pooled using SequalPrepTM Normalization Plate Kit (ThermoFisher) according to the manufacturer’s instructions. The library was purified using Axygen AxyPrep Mag PCR Clean-UpKit (Fisher Biotech) as per the manufacturer’s instructions. Concentration and quality of the pooled library were checked with Qubit and the library size was assessed in an Agilent 2200 TapeStation instrument. The Agencourt AMPure XP Bead Clean-up kit was used on the pool to reduce/remove the presence of primer dimers. The library pool was sequenced on the MiSeq using a MiSeq Reagent Kit v3 with a 2 × 300 bp run format, using default run parameters including adaptor trimming. For these runs, custom primers were added to the reagent cartridge for Read1, Index, and Read2.

### Bioinformatics analysis

The reads were analysed according to MiSeq [25] protocol using mothur (v1.39.5) [26]. Briefly, the reads were quality filtered and assigned to their respective samples. After trimming samples with length between 405 and 495 bp were retained. Samples with homopolymers longer than 8 bp were removed. Chimera.vsearch script in mothur [27] was used to remove chimeric sequences and SILVA reference alignment (v132) [27] was used to align and classify the sequences. The lineages not targeted by the primer pair i.e. archaea, chloroplast, eukaryote, mitochondria and unknown sequencing errors were removed. The sequences were grouped into OTUs based on 97% similarity using OptiClust algorithm [28] and subsampled based on the sample with the lowest number of sequences. ZymoBIOMICS Microbial Community Standards was used as control in each sequencing run to assess sequencing error. Krona [29] was used to create interactive OTU plots from the subsampled data and mothur_krona_XML.py script [30] was used to generate OTU richness plot. For the generation of diversity plots, OTUsamples2krona.sh script [31] was used by providing reformatted mothur biom file.

### Statistical analysis

The *Salmonella* Typhimurium load in organs was analysed in GraphPad Prism (version 9.0.2) considering treatment as main effect. For the quantification of *Salmonella* Typhimurium from faeces, treatment and time point were considered as main effects. The level of significance was determined by Fisher’s protected least significant difference (PLSD) at *P* < 0.05. The gut microbiota data were analysed in Calypso software [32] using ANOVA and diversity analysis (Shannon index). The total sum normalised method was used to transform the data. The visualisation of data (q-PCR and comparative analysis of gut microbiota) was performed in GraphPad Prism (version 9.0.2).

## Results

### Mortality and clinical signs of salmonellosis

There were no visible clinical signs in the infected chickens. There was no mucoid faeces or diarrhoea observed in any of the treatment groups. The swabs collected from the incubators were *Salmonella* negative. All the chicks were tested for *Salmonella* negative before the challenge. Mortality (*n* = 3) was observed in the PC group in week 1 after infection. However, no clinical sings or post-mortem lesions were observed in the birds.

### *Salmonella* Typhimurium detection in faecal samples

A total of 584 faecal samples were collected over the experimental period and monitored for *Salmonella* Typhimurium. All the chickens from the *Salmonella* negative control groups (NC_CORT and NC_FW) were negative and all the chickens from the *Salmonella* infected groups (INF_CORT, INF_PBS, PC and INF_FW) were positive from the time of post-infection until the end of the trial.

### *Salmonella* Typhimurium quantification from tissue samples

All the tissue samples collected from the negative control groups were negative for *Salmonella* Typhimurium. Liver and spleen collected from the infected groups did not show any significant difference in log_10_ CFU count of *Salmonella* Typhimurium (INF_CORT, INF_PBS, PC and INF_FW). There was no significant difference in log_10_ CFU count of *Salmonella* Typhimurium in caecum and colon in all infected groups (Additional file 1: Table S1). In the ileum, the significant difference in the bacterial count was observed only between the group INF_PBS and PC (*P* = 0.0487). Negative samples after direct plating were enriched in buffer peptone water and RVS. After enrichment, organs (liver, spleen and ileum) showed significant differences in *Salmonella* detection level (Additional file 1: Table S1). Post enrichment *Salmonella* positive rate in liver in group INF_PBS was significantly lower (*P* = 0.0346) as compared to the PC group. The spleen samples from INF_CORT treatment group showed significantly low *Salmonella* positive samples as compared to INF_PBS (*P* = 0.0268) and INF_FW (*P* = 0.0451) groups. Furthermore, *Salmonella* positive spleen samples post enrichment in the INF_PBS group was significantly lower (*P* = 0.0491) compared to the INF_FW group.

### Enumeration of *Salmonella* Typhimurium in faecal samples by quantitative PCR

*Salmonella* Typhimurium load (log_10_ CFU/gram) was detected using q-PCR from faecal samples collected at different time points. The cut-off Ct value was 31*.*

**Fig. 1:**
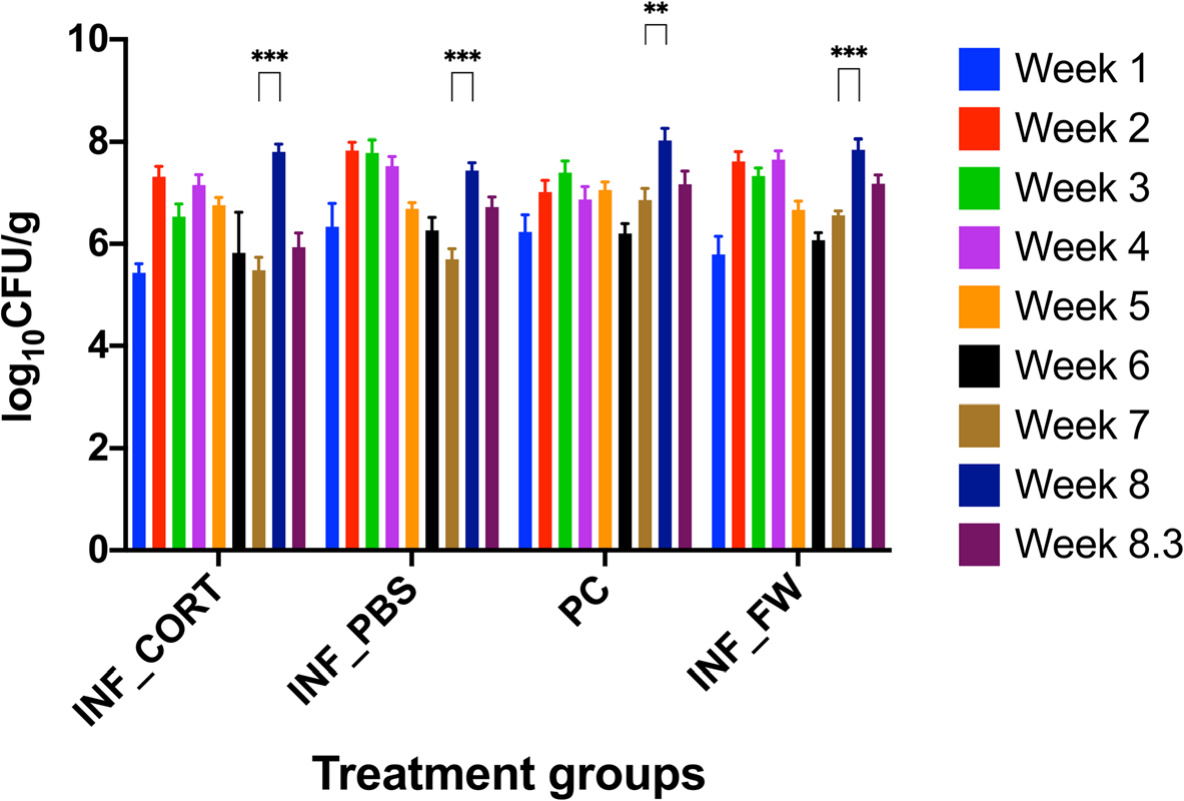
Bacterial load quantified using qPCR from faecal samples collected from chickens infected with *Salmonella* Typhimurium from week 1 to week 8 post infection. Groups INF_ CORT and INF_PBS received intramuscular injection of corticosterone or PBS, while group INF_FW had feed withdrawal which showed a significant (*P* < 0.0001) increase in *Salmonella* Typhimurium count at week 8 post infection. Group PC also showed a significant increase (*P* = 0.0005) in *Salmonella* Typhimurium count at week 8 post infection. *Salmonella* Typhimurium was detected using TSR3 gene specific primer pair and the data are presented as log_10_ CFU ± S.E.M. The data were visualised in GraphPad Prism v.8.0.0. Asterisks (** and ***) show *P* values at 0.001 and 0.0001 respectively

Samples were collected from all 6 groups at week 1, 2, 3, 4, 5, 6, 7, 8, 8.3 post infection. The samples collected from the negative control groups (NC_CORT and NC_FW) were negative for *Salmonella* Typhimurium throughout the experiments. These results were confirmed by testing negative samples by gel electrophoresis. Significant effect of time (*P* < 0.0001) and treatment groups (*P* < 0.0001) was observed on *Salmonella* Typhimurium load in faecal samples (Fig. 1).

After the injection and feed withdrawal (at week 8post infection), no significant differences in log_10_ CFU of *Salmonella* Typhimurium were observed between the groups except in between INF_PBS vs PC (*P* = 0.0292). A significant increase in *Salmonella* Typhimurium shedding was observed within the groups at week 8 compared to week 7 post infection in INF_CORT (*P* < 0.0001), INF_PBS (*P* < 0.0001), PC (*P* = 0.0005) and INF_FW (*P* < 0.0001) treatment groups.

### *Salmonella* Typhimurium affects the abundance and diversity of gut microbiota at week 1 post infection

In total, 360 DNA samples were sequenced to understand the effects of *Salmonella* Typhimurium infection on gut microbiota composition. In this trial, we did not observe significant bird to bird variation in gut microbiota composition. Therefore, the data from infected groups (INF_CORT, INF_PBS, PC and INF_FW) and negative control groups (NC_CORT and NC_FW) for week 1 post infection and up to week 7 post infection were combined for analysis and presentation.

**Fig. 2:**
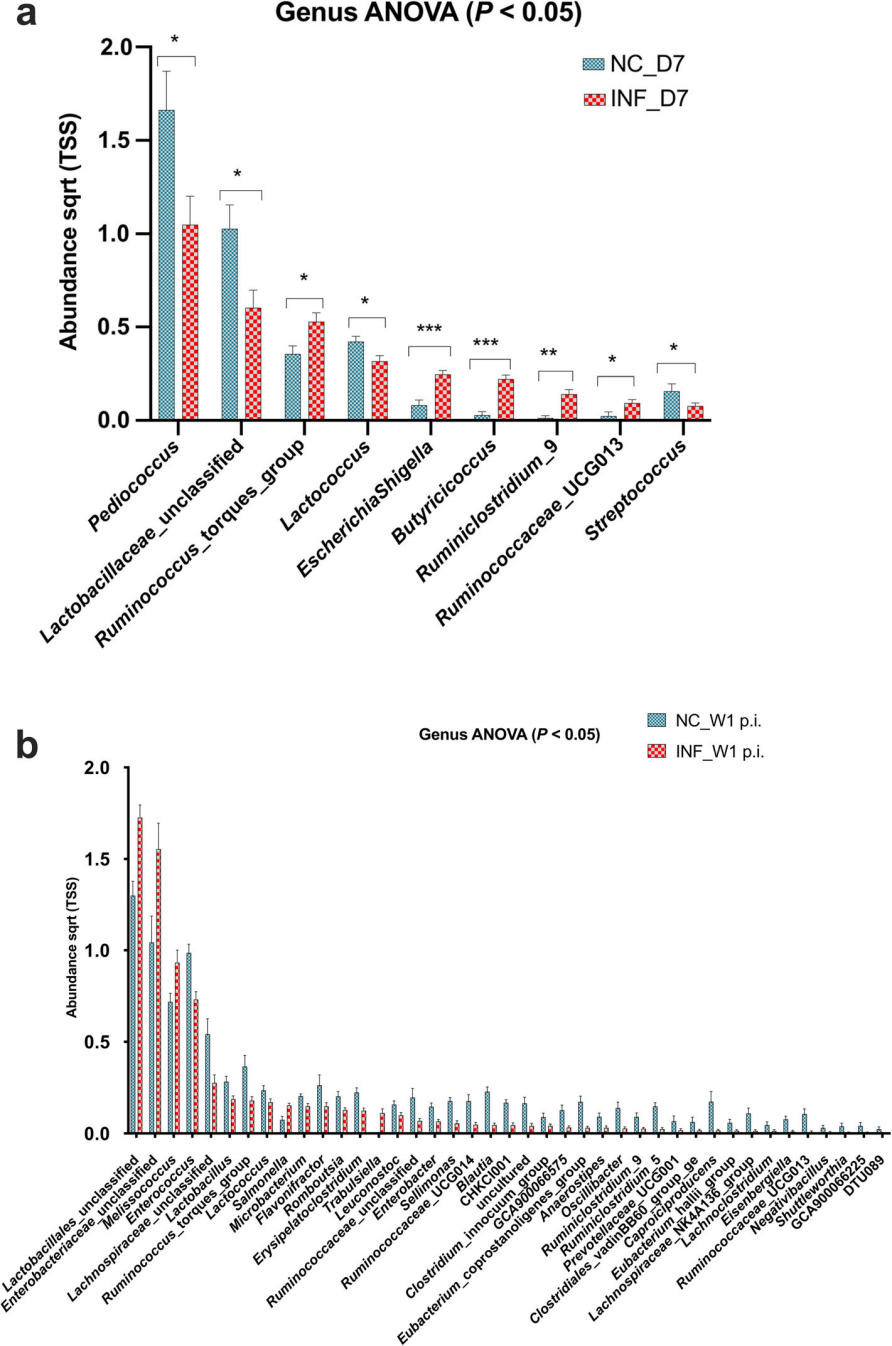
The relative abundance of different genera in the negative control (NC) and *Salmonella* Typhimurium infected (INF) groups at d 7 post hatch and week 1 post infection. **a** The relative abundance of significant genera at d 7 post hatch. **b** The abundance of significant genera at week 1 post *Salmonella* Typhimurium infection. The relative abundance levels were calculated in Calypso software and the data were visualised in GraphPad Prism v.8.0.0. Asterisks (*, ** and ***) show *P* values at 0.01, 0.001 and 0.0001 respectively

The comparative analysis of gut microbiota between the infected (INF) and the negative control (NC) groups showed significant differences in the abundance of various genera week 1 post infection compared to d 7 (Fig. 2a, b). At week 1 post infection, the infected group showed significantly (*P* < 0.05) higher abundance of genera including *Trabulsiella*, *Melissococcus*, *Lactobacillales_* unclassified and *Enterobacteriaceae*_ unclassified compared to the negative control group. The *Salmonella* Typhimurium infected group showed a significantly lower abundance of genera including *Ruminococcus*_torques_group, *Ruminococcaceae*_unclassified, *Ruminococcaceae*_UCG014, *Lachnospiraceae*_unclassified, *Enterococcus* and *Blautia* compared to the negative control group. The infected groups showed a significant reduction in genera *Ruminococcus*_torques_group and *Ruminococcaceae*_UCG013 at week 1 post infection as compared to d 7 (Fig. 2a, b).

**Fig. 3:**
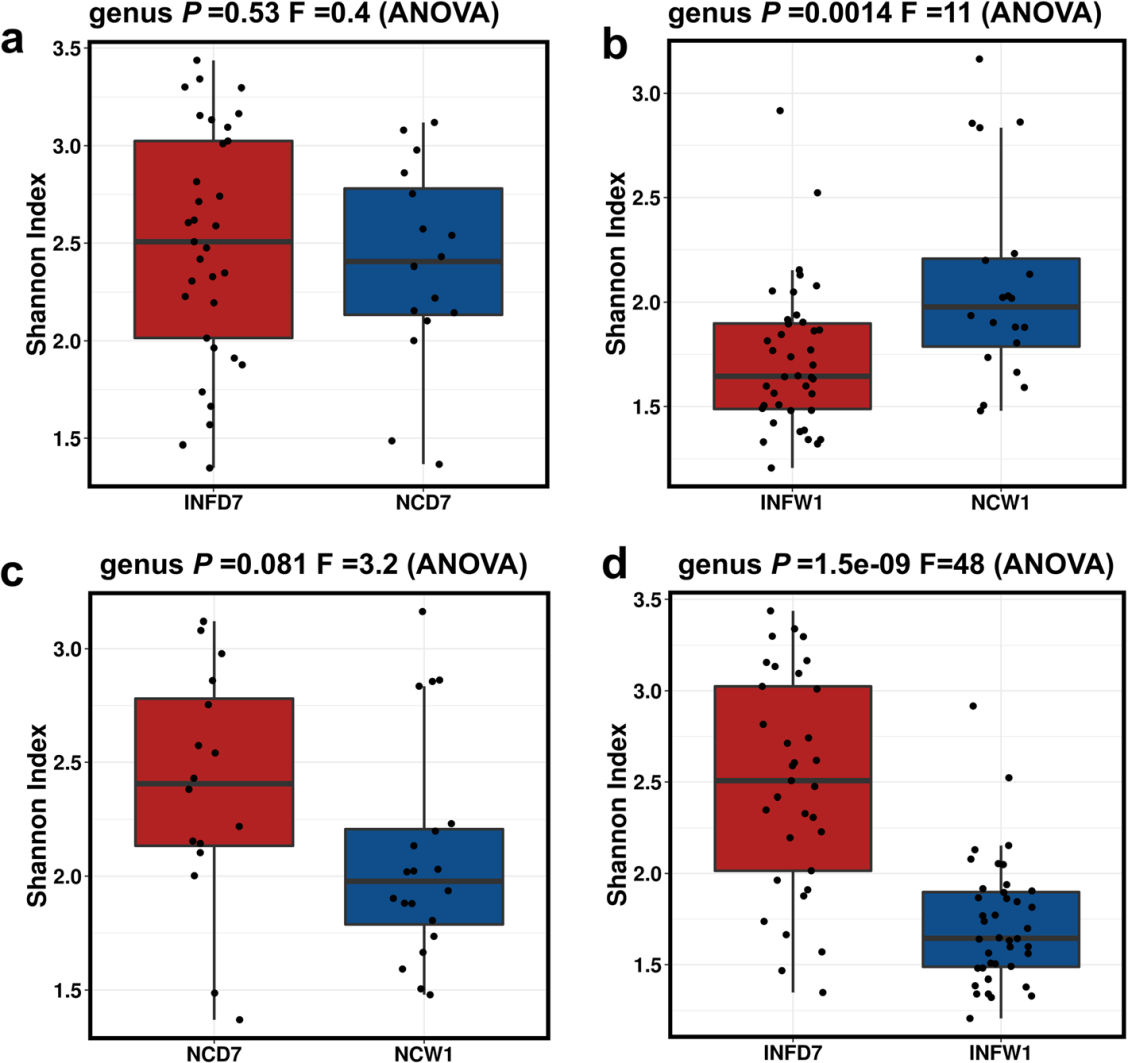
Alpha diversity of gut microbiota at genus level affected by *Salmonella* Typhimurium infection. **a** Alpha diversity between negative control (NC) and *Salmonella* Typhimurium infected (INF) groups at d 7 post hatch. **b** Alpha diversity between NC and INF groups at week 1 post infection. **c** Alpha diversity of NC group at d 7 post hatch and week 1 post infection. **d** Alpha diversity of INF group at d 7 post hatch and week 1 post infection. Alpha diversity was measured by Shannon index in Calypso software

On d 7, the alpha diversity of microbial communities within individual samples of the NC and INF was not significantly different (Fig. 3a) as compared to week 1 post infection (Fig. 3b), where the alpha diversity in infected groups reduced significantly (*P* = 0.0014). Alpha diversity analysis within the groups between d 7 and week 1 post-infection showed no significant differences (*P* = 0.081) in the negative control group (Fig. 3c); however, the infected group showed a significant reduction (*P* = 1.5e-09) in alpha diversity (Fig. 3d).

### Abundance and diversity of gut microbiota up to week 7 post infection in negative control and *Salmonella* Typhimurium infected groups

**Fig. 4:**
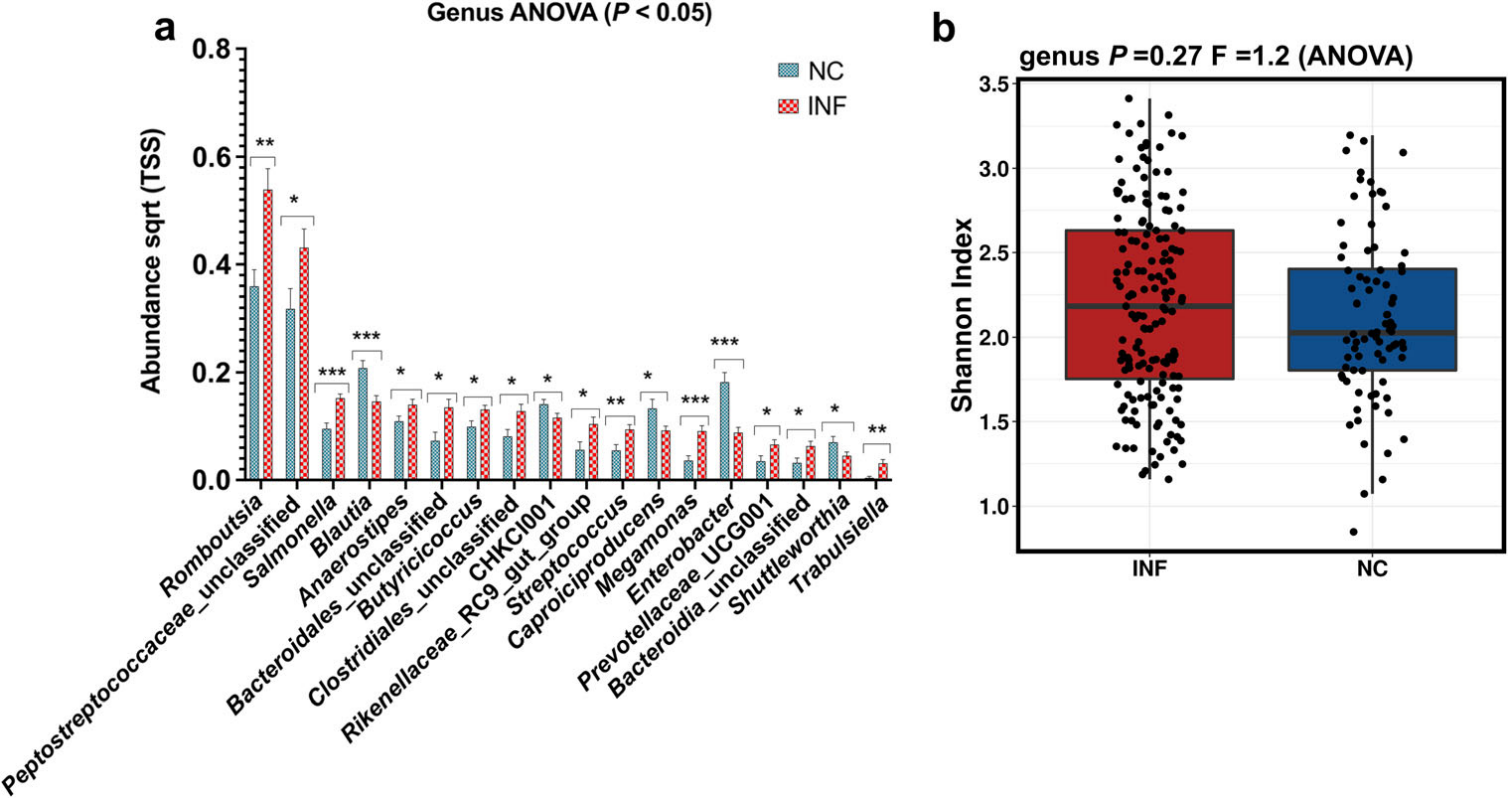
Relative abundance and diversity of gut microbiota. **a** The abundance of different genera in the negative control (NC) and *Salmonella* Typhimurium infected (INF) treatment groups from week 1 to week 7 post infection. **b** Comparative analysis of alpha diversity between NC and INF groups from week 1 to week 7 post infection using Shannon index. The relative abundance levels were calculated in Calypso software and the data were visualised in GraphPad Prism v.8.0.0. Asterisks (*, ** and ***) show *P* values at 0.01, 0.001 and 0.0001 respectively

The gut microbiota from the negative control group and *Salmonella* Typhimurium infected group was compared from week 1 to week 7 post infection to analyse the abundance and diversity at the genus level (Fig. 4a). In *Salmonella* Typhimurium infected group, the abundance of microbial communities including *Streptococcus*, *Romboutsia*, *Rikenellaceae*_RC9_gut_group, *Peptostreptococcaceae*_unclassified and *Butyricicoccus* was significantly higher compared to the negative control group (Fig. 4a). In negative control groups, abundance of the genus including *Enterobacter,* CHKCI001, C*aproiciproducens* and *Blautia* were significantly higher as compared to *Salmonella* Typhimurium infected group (Fig. 4a). Alpha diversity measured by Shannon index did not show any significant difference in the overall gut microbiota up to week 7 post infection between the infected and negative control groups (Fig. 4b).

### Effect of the injection and feed withdrawal on gut microbiota diversity and Proteobacteria-Firmicutes ratio

**Fig. 5:**
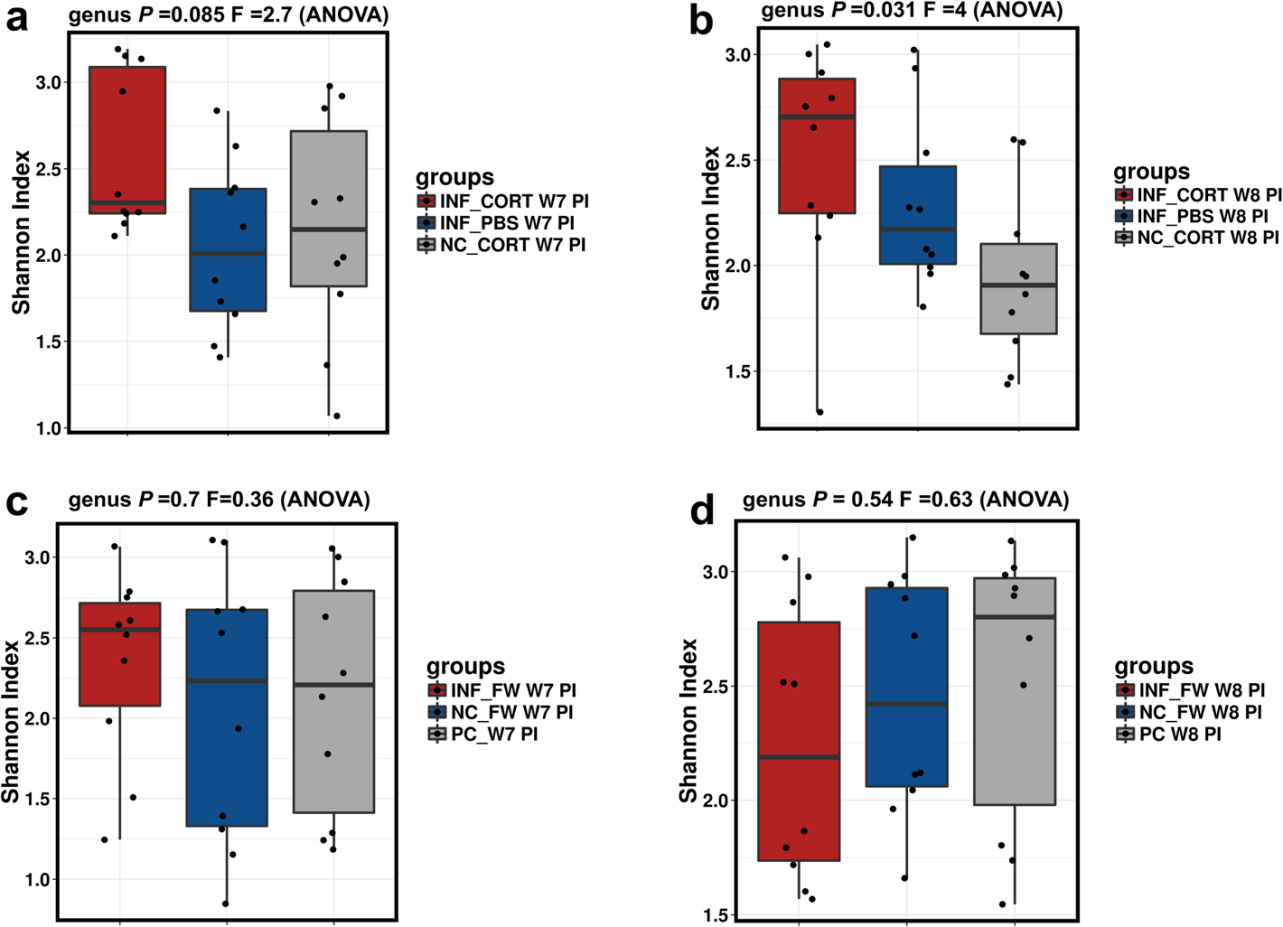
Alpha diversity of gut microbiota at genus level affected by *Salmonella* Typhimurium infection, intramuscular injection and feed withdrawal. **a** Alpha diversity of gut microbiota before the intramuscular injection (week 7 *Salmonella* Typhimurium post-infection). **b** Alpha diversity of gut microbiota after the induction of intramuscular injection (week 8 *Salmonella* Typhimurium post-infection). **c** Alpha diversity of gut microbiota before the feed withdrawal (week 7 *Salmonella* Typhimurium post-infection). **d** Alpha diversity of gut microbiota after the feed withdrawal (week 8 *Salmonella* Typhimurium post-infection). Alpha diversity was measured by Shannon index in Calypso software. For treatment group details, refer to Table 1

To understand the effects of intramuscular injection and feed withdrawal on the composition of gut microbiota, data obtained at week 7 and week 8 were compared within the respective treatment groups. Compared to week 7 (Fig. 5a), intramuscular injections significantly affected the alpha diversity of gut microbiota among the negative control and *Salmonella* Typhimurium challenged groups at week 8 of chickens age (Fig. 5b). Compared to week 7 (Fig. 5c), feed withdrawal did not significantly (*P* = 0.54) impact the alpha diversity of gut microbiota within and between the groups (Fig. 5d).

**Fig. 6:**
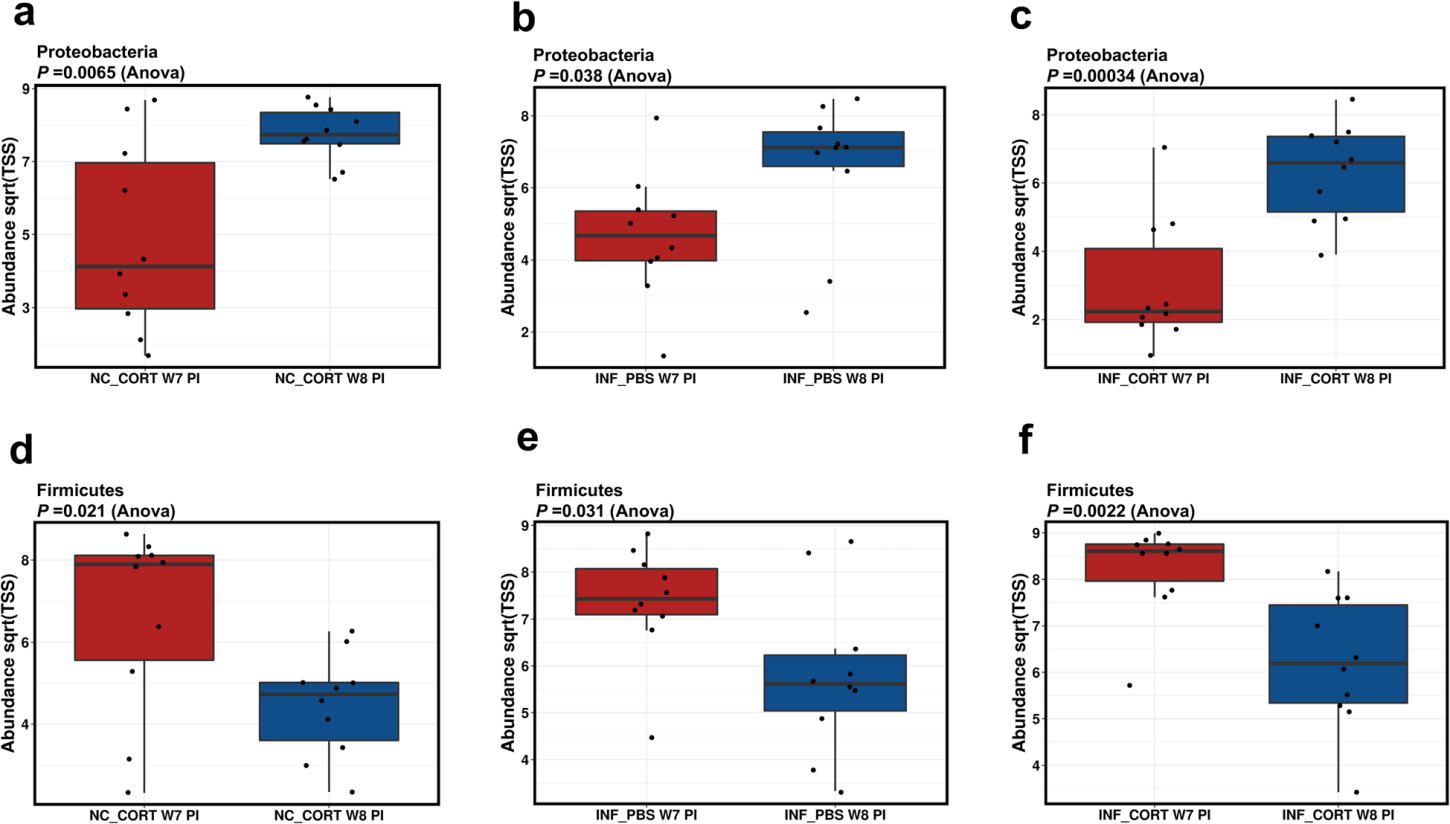
Alpha diversity of Proteobacteria and Firmicutes affected by intramuscular injection. **a** Proteobacteria in negative control and corticosterone injected treatment group (NC_CORT) at week 7 and 8 post *Salmonella* infection. **b** Proteobacteria in *Salmonella* Typhimurium infected treatment (INF_PBS) group at week 7 and 8 post-infection. **c** Proteobacteria in *Salmonella* Typhimurium infected and corticosterone injected treatment group (INF_CORT) at week 7 and 8 post *Salmonella* infection. **d** Firmicutes in negative control and corticosterone injected treatment group (NC_CORT) at week 7 and 8 post *Salmonella* infection. **e** Firmicutes in *Salmonella* Typhimurium infected treatment group (INF_PBS) at week 7 and 8 post *Salmonella* infection. **f** Firmicutes in *Salmonella* Typhimurium infected and corticosterone injected treatment group (INF_CORT) at weeks 7 and 8 post *Salmonella* infection. NC_CORT and INF_CORT received intramuscular injection of corticosterone, while group INF_PBS received intramuscular injection of PBS

**Fig. 7:**
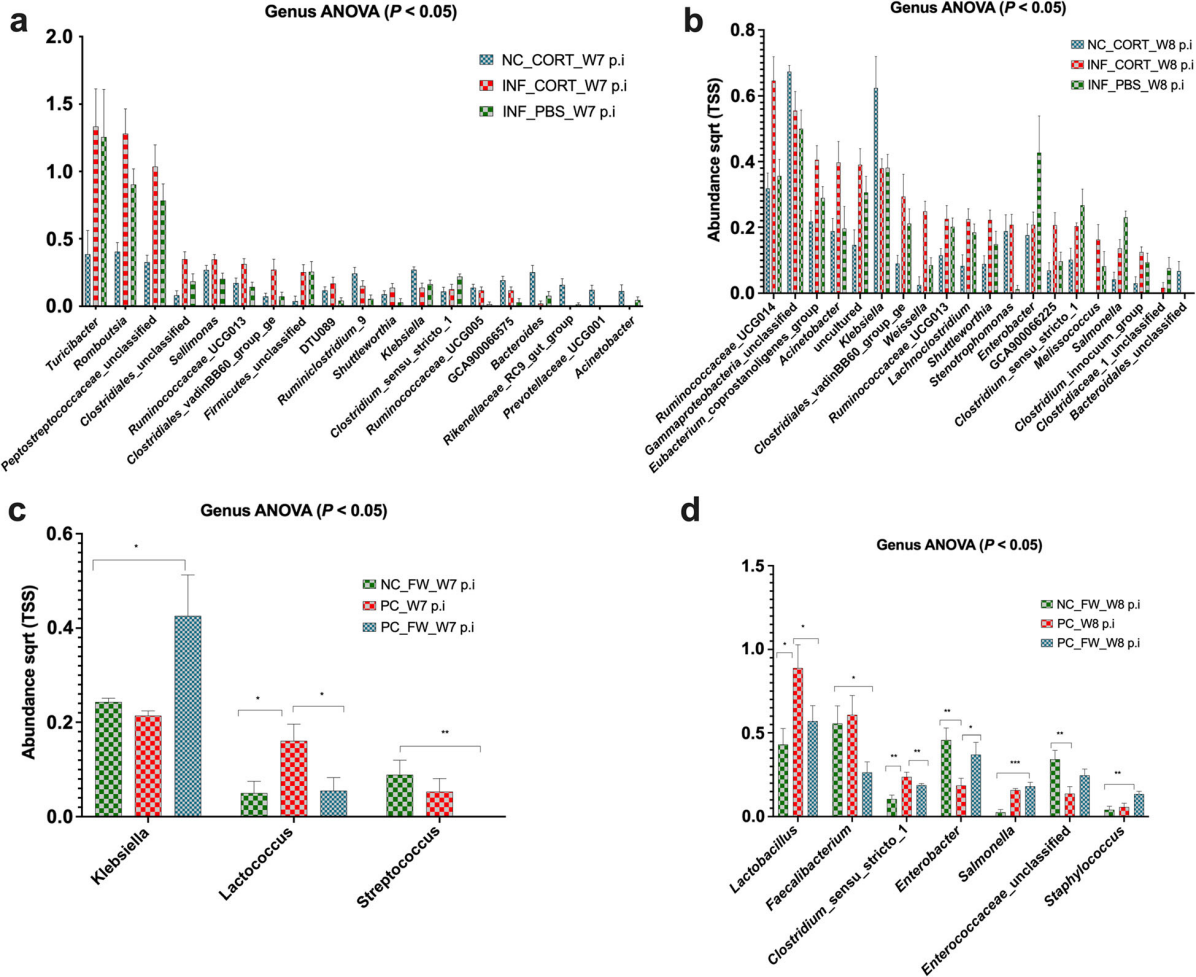
The relative abundance of microbial communities at genus level affected by intramuscular injection and feed withdrawal. **a** The relative abundance of gut microbiota before the induction of intramuscular injection (week 7 *Salmonella* Typhimurium post-infection). **b** The relative abundance of gut microbiota after the induction of intramuscular injection (week 8 *Salmonella* Typhimurium post-infection). **c** The abundance of gut microbiota before the induction of feed withdrawal (week 7 *Salmonella* Typhimurium post-infection). **d** The abundance of gut microbiota after the induction of feed withdrawal (week 8 *Salmonella* Typhimurium post-infection). For treatment group codes, refer to Table 1

After the intramuscular injection, significant differences were observed in the abundance of phyla Proteobacteria and Firmicutes in groups NC_CORT, INF_CORT and INF_PBS (Fig. 6a–f). The abundance of Proteobacteria increased significantly at week 8 post-infection in NC_CORT (mean = 7.76), INF_CORT (mean = 6.33), INF_PBS (mean = 6.53) as compared to week 7 post infection NC_CORT (mean = 4.88), INF_CORT (mean = 3), PC_PBS (mean = 4.66). The abundance of Firmicutes significantly decreased at week 8 in NC_CORT (mean = 4.47), INF_CORT (mean = 6.21), INF_PBS (mean = 5.8) as compared to week 7 in NC_CORT (mean = 6.61), INF_CORT (mean = 8.22), INF_PBS (mean = 7.36) groups. The significant differences in group NC_CORT, INF_CORT and INF_PBS in gut microbiota at the genus level were also observed (Fig. 7a, b). A significantly higher abundance of microbial communities including *Klebsiella* and *Gammaproteobacteria_*unclassified was observed in the non-infected corticosterone injected group (NC_CORT) at week 8 post- infection (Fig. 7b). In the *Salmonella* Typhimurium infected and corticosterone injected group (INF_CORT), the abundance of genera including *Ruminococcaceae*_UCG014, *Ruminococcaceae*_UCG013, Melissococcus, *Lachanoclostridium* and *Acinetobacter* was significantly higher at week 8 post infection. In feed withdrawal groups (NC_FW, PC and INF_FW), no significant differences were observed in the gut microbiota after feed withdrawal at phylum level; however, the gut microbiota in feed withdrawal group showed a significant difference in abundance at genera level (Fig. 7c, d) The abundance levels of genera *Lactobacillus* and *Faecalibacterium* were higher in the *Salmonella* infected group (PC).

## Discussion

The present study was conducted to understand the effects of intramuscular injection and feed withdrawal on *Salmonella* Typhimurium shedding and gut microbiota composition in pullets. In an earlier infection trial, chicks inoculated with *Salmonella* Typhimurium showed persistence in caeca until the age of week 7 post infection. However, the chicks were inoculated with 10^6^ to 10^7^ CFU of *Salmonella* Typhimurium [33]. In this study, we observed that all the *Salmonella* Typhimurium inoculated birds were positive until the end of the trial i.e., up to week 8 post infection despite a low challenge dose of *Salmonella* Typhimurium (10^3^ CFU). This could suggest that *Salmonella* Typhimurium can colonise and shed persistently in faeces at least up to week 8 even at low doses of infections. Some studies reported the detection of *Salmonella* Typhimurium from infected hens up to week 15 [34] and week 16 [35] post infection; however, the birds in those studies were inoculated with 10^9^ CFU of *Salmonella* Typhimurium and the studies were conducted in older birds. In an earlier study 10^3^ CFU was used to infect chicks at d 7 which reported colonisation of *Salmonella* Typhimurium in caeca, liver and spleen. However, this study was conducted for 1 week and did not focus on shedding pattern in faecal samples [16]. During this study, birds did not show any clinical signs after *Salmonella* Typhimurium infection. This finding is in agreement with previous studies [16, 36].

In this study, we did not find any significant differences in bacterial load in the spleen and liver from the *Salmonella* Typhimurium positive groups after direct plating. These findings were in contrast to the earlier studies where a significant *Salmonella* load was detected in the liver and spleen [8, 16, 35]. The caeca and colon did not show significant differences in *Salmonella* load between the *Salmonella* infected groups. The negative ileum samples for *Salmonella* Typhimurium showed significant differences in *Salmonella* load on enrichment in RVS except between group INF_PBS and PC. This can be attributed to the potential stress induced by intramuscular PBS injections in the INF_PBS group. An earlier study has reported that stress results in increase *Salmonella* shedding in animals [37]. Earlier *Salmonella* colonisation studies were focused on the caecum, liver and spleen [8, 16, 35]. However, our study suggests that ileum and colon could also be the potential sites for colonisation of *Salmonella* Typhimurium in chickens.

The significant increase in *Salmonella* count in faeces at week 8 post-infection in the groups INF_CORT, INF_PBS, PC and INF_FW groups can be co-related to the potential stress caused by intramuscular injection and feed withdrawal induced 24 h prior. Interestingly, the *Salmonella* count in faeces after intramuscular injection did not show any significant differences in INF_CORT and INF_PBS groups, which was not expected. However, the increased shedding in INF_CORT group (7.805 ± 0.151) was 2.323 log CFU higher at week 8 as compared to increased shedding in INF_PBS (7.439 ± 0.149) where it was higher by 1.744 log CFU at week 8. It is unclear why INF_PBS group showed increase in shedding although it does support the notion that potential stress induced by handling or intramuscular injection, increased *Salmonella* Typhimurium shedding in faeces. If a *Salmonella* infected flock is to be vaccinated for other diseases, the increase in bacterial shedding in faeces is to be expected, and hence, the personal hygiene of the bird handling crew and farm managers is important to reduce the risk of zoonosis. The *Salmonella* count in faecal samples did not show a significant difference between groups INF_FW which was subjected to feed withdrawal and PC. Although there was no significant difference in both the groups, increase in *Salmonella* Typhimurium shedding in INF_FW group (7.845 ± 0.207) was 1.284 log CFU higher at week 8 as compared to the PC group (8.025 ± 0.228) where it was higher by 1.164 log CFU at week 8. All four groups showed significant increase in *Salmonella* Typhimurium shedding at week 8 post infection as compared to week 7 post infection. These results could not be compared as there are no such studies conducted on chickens. A study monitoring *Salmonella* Typhimurium load in pigs after feed withdrawal and transport stress showed an increased *Salmonella* Typhimurium load in the gut; however, in that study feed withdrawal was for 24 h and instead of corticosterone, dexamethasone was used because of its longer half-life as compared to corticosterone to induce stress [37]. The current study demonstrated that the intramuscular injections and feed withdrawal resulted in increased *Salmonella* count in faecal samples. However, this study did not investigate the effect of intramuscular injections and feed withdrawal on host corticosterone levels in chickens. Thus, further studies are needed to understand the effect of intramuscular injections and feed withdrawal on corticosterone levels in chickens. Although, in an earlier study in mice, handling and intraperitoneal saline injection have been co-related with increased plasma corticosterone levels [38]. The increased faecal *Salmonella* count could be an indicator of recrudescence of *Salmonella* Typhimurium; however, multiple studies are required to understand the actual mechanism of recrudescence in chickens.

In this study, the gut microbiota showed significant changes in diversity due to *Salmonella* Typhimurium infection. The alpha diversity of gut microbiota was estimated by using the Shannon index that measures the richness and evenness in the distribution of species in the given gut microbiota community [32]. Furthermore, the *Salmonella* Typhimurium infected group showed a significant reduction in the alpha diversity at week 1 post infection. In growing chicks, normally the diversity of gut microbiota increases with age [9]. In the current study, *Salmonella* Typhimurium infection was negatively co-related with *Ruminococcus_torques, Ruminococcaceae_*unclassified, Runimoclostridium_9, Oscillibacter, Lactobacillus, Lachnospiraceae_unclassified, Flavonifractor, Erysipelatoclostridium, Eisenbergiella, Caproiciproducens and *Blautia*. The reduction in these genera in infected groups after 1 week post-infection could suggest that not all the bacterial genera can compete with *Salmonella* Typhimurium infection and leads to the reduction in their abundance. These findings are partially supported by an earlier study that analysed the effect of *Salmonella* Typhimurium on gut microbiota modulation in pullets [8]. However, in the current study, the abundance of many different genera was also affected. This could be attributed to bird-to-bird differences as well as age which is an important influencing factor for gut microbiota [39]. Further, in this study chicks were challenged at d 7 compared to 18-week-old pullets which had resilient and developed microbiota [9]. Thus, it could be assumed that *Salmonella* Typhimurium infection at an early age could have lingering effects on gut microbiota composition. However, further studies are required to understand this hypothesis. *Ruminococcaceae* (*Ruminococcus_*torues, *Ruminococcaceae_*unclassified, *Ruminoclostridium_9*) are natural inhabitants of the chicken gut and play a key role in a breakdown in complex carbohydrates [8]. *Ruminococcaceae* and *Lachnospiraceae* (*Lachnospiraceae_*unclassified, *Eisenbergella*) are major producers of butyric acid which is a preferred substrate of intestinal epithelial cells [9]. *Blautia* is the most abundant member of gut microbiota responsible for the production of butyric and acetic acid. The lower abundance of *Blautia* has been co-related to obesity, diabetes, liver cirrhosis and rectal cancer in humans [40]. Reduction of these beneficial genera at week 1 post infection in *Salmonella* infected group can have adverse effects on health and also affect the performance of the birds.

The *Salmonella* infection also reduced abundance of *Falvinofractor* and *Erysipelitoclostridium* in infected groups*. Flavinofractor* is a known opportunistic pathogen known to cause infections in immunocompromised patients [41]. *Erysipelitoclostridium* has been co-related to Crohn’s disease and *Clostridium difficile* infections in humans [42]. Thus, from the available data, it could be concluded that *Salmonella* Typhimurium infection at an early age disrupts the normal gut microbiota structure which could adversely affect overall health and growth of the birds.

In this study, abundance of *Melissococcus* and *Enterabacteriaceae_*unclassified increased in *Salmonella* Typhimurium infected groups. Both these genera belong to family *Enterobacteriaceae*, which contains known pathogens. *Melissococcus pluton* is also known to cause European foulbrood in honeybees [43].

The analysis of gut microbiota composition between week 1 post infection to week 7 post infection showed increased abundance of *Streptococcus*, *Peptostreptococcaceae_*unclassified, *Romboutsia*, *Megamonas, Anaerostipes* in gut microbiota. *Streptococcus* and *Peptostreptococcaceae_*unclassified are known pathogenic genera. An increase in abundance of *Rombutsia* was in contrast to the earlier report which suggested that *Salmonella* Typhimurium load was negatively co-related to its abundance [8]. Genus *Romboutsia* plays role in the synthesis of vitamins and cofactors and fermentation of single amino acids [44]. An earlier study suggested that genus *Anaerostipes* could have a protective effect against colon cancer in humans [45]. However, more studies are required to understand its role in chickens. Further, the alpha diversity analysis using the Shannon index between the infected and negative control groups did not show any significant differences. This data suggests that over the period (up to week 7 post infection) gut microbiota could have recovered from disruption caused by *Salmonella* Typhimurium infection.

The feed withdrawal did not show any significant difference in diversity of the gut microbiota; however, the intramuscular injection (of both PBS and corticosterone) reduced the diversity of gut microbiota, which has been documented earlier [46]. Furthermore, stress induced in groups (NC_CORT, INF_CORT and INF_PBS) by intramuscular injection showed a significant decrease in phylum Firmicutes and a significant increase in phylum Proteobacteria at week 8 post infection. The feed withdrawal did not result in any significant variation in both the phyla. There are limited studies in chickens that assessed the effect of different stressor on the host gut microbiota. The data available in chickens is focused on effect of heat stress on gut microbiota in chickens [47]. A study in layers showed that exposure to high temperature significantly reduced abundance of phylum Firmicutes [48]. However, these results are contradicted by studies in broilers and in Shaoxing ducks in which exposure to high temperature showed increased abundance of the phylum Firmicutes [49, 50]. A study in humans suggest that the effect of stressors on gut microbiota vary across stressors [51]. Layers are exposed to multiple potential stressors throughout their life span such as vaccination, transport, beak trimming, change in feed and extreme weather in free range production systems. Thus, studies are required focusing on effect of different stressors on gut microbiota in chickens. Although the immune functions were not the focus of this study, it is known that stress adversely impacts the immune functions by suppression of immunity, altering the intestinal morphology that intern can affect the gut microbiota in the host [49, 52–54]. The results in groups INF_CORT and INF_PBS also co-relate with a significant increase in faecal *Salmonella* Typhimurium count at week 8 post infection. Earlier studies reported that stress (induced by corticosterone) leads to replication of *Salmonella* Typhimurium in pigs by modulating the immune system [6]. This replication is driven by the _*SCS*_*A* gene which under the influence of cortisol induces macrophage cytoskeletal rearrangement [6]. This facilitates the intracellular replication of *Salmonella* Typhimurium in macrophages [6]. The mechanism of *Salmonella* replication in chickens under stress is unknown. The stress associated with feed withdrawal and transport has been shown to increase caecal carriage of *Salmonella* in broilers [55, 56]. Further, the undetected infections in pigs leads to the continues shedding of *Salmonella* Typhimurium due to stress caused by transportation and feed withdrawal [57–59] however, it can be assumed that potential stress due to intramuscular injections could have increased *Salmonella* shedding in this study. The increase in the count of *Salmonella* could have contributed to an increased abundance of phylum Proteobacteria. This could be because “like will to like” concept proposed by Stretcher et al. which may help to understand the increase in closely related bacterial species in the gut [60]. Phylum Proteobacteria includes the *Enterobacteriaceae* family which contain know enteric pathogens like *Shigella* and *Escherichia coli* [7]. Thus, increased abundance of phylum Proteobacteria could increase susceptibility to the infection [7]. The significant increase in phylum Proteobacteria can affect the Proteobacteria-Firmicutes ratio which could lead to dysbiosis in the gut [60]. Significant decrease in phylum Firmicutes after intramuscular injections in *Salmonella* infected group was not only the result of the infection but can also be the predisposing factor for the growth of *Salmonella*. Phylum Firmicutes contains families like *Ruminococcaceae* and *Lachnospiraceae* which are reported to be inversely proportional to the abundance of family *Enterobacteriaceae* [7]. These families produce butyric acid, which is a short chain fatty acid that down regulates *Salmonella* Pathogenicity Island 1 expression. This could lead to a reduced invasion capability of bacteria in a host [61].

## Conclusions

In conclusion, our findings suggest that exposure to relatively lower dose of *Salmonella* can lead to persistent shedding in pullets. Furthermore, our data suggest that colon and ileum could be potential anatomical sites for colonisation and replication of *Salmonella* Typhimurium in chickens; however, detailed studies are required to test this hypothesis. The intramuscular injections mimicking field practises (such as vaccinations), and handling (shifting) and feed withdrawal could increase *Salmonella* shedding in infected or carrier pullets. The study also concludes that *Salmonella* Typhimurium infection at an early age significantly reduces the diversity of gut microbiota and disrupts the microbiota composition. Furthermore, intramuscular injections altered the gut microbiota composition by significantly changing Proteobacteria- Firmicutes ratio, which could increase the risk of dysbiosis.

## Supplementary Information

**Additional file 1: Table S1.** Comparative analysis of *Salmonella* Typhimurium positive tissue samples between direct plating and post enrichment *Salmonella* Typhimurium load in organ at the time of cull.

## Data Availability

The 16S rRNA sequence data are available from the NCBI SRA under the BioProject accession number PRJNA647923.
