# Supplementary material for: Understanding the effects of intramuscular injection and feed withdrawal on Salmonella Typhimurium shedding and gut microbiota in pullets
Source: J Anim Sci Biotechnol. 2021 Jun 4;12:78. doi: 10.1186/s40104-021-00597-9 (PMC8178826; doi:10.1186/s40104-021-00597-9)
Supplement: Supplementary file 1 — Additional file 1: Table S1. Comparative analysis of Salmonella Typhimurium positive tissue samples between direct plating and post enrichment Salmonella Typhimurium load in organ at the time of cull. [file 40104_2021_597_MOESM1_ESM.docx]

| Sr. No | Organs | **INF_CORT** | | **INF_PBS** | | **PC** | | **INF_FW** | |
| --- | --- | --- | --- | --- | --- | --- | --- | --- | --- |
|  |  | **Direct plating** | **Post-enrich** | **Direct plating** | **Post-enrich** | **Direct plating** | **Post-enrich** | **Direct plating** | **Post-enrich** |
| 1 | **Liver** | 0/13 | 2/13 | 1/13  (1.83±0.00) | 1/12 | 0/11 | 1/11 | 3/12  (1.98±0.08) | 1/9 |
| 2 | **Spleen** | 0/13 | 1/13 | 1/13  (2.649± 0.0) | 1/12 | 0/11 | 3/11 | 2/12  (1.54±0.00) | 1/10 |
| 3 | **Ileum** | 5/13  (3.22±0.26) | 3/8 | 4/13  (4.077±0.25) | 6/9 | 6/11  (2.54±0.25) | 0/5 | 3/12  (4.44±0.00) | 1/9 |
| 4 | **Caeca** | 13/13  (6.21±0.16) | 13/13 | 13/13  (6.83±0.12) | 13/13 | 11/11  (6.33±0.27) | 11/11 | 12/12  (6.08±0.57) | 12/12 |
| 5 | **Colon** | 8/13  (4.52±0.39) | 5/5 | 13/13  (4.51±0.29) | 13/13 | 8/11  (4.07±0.44) | 2/3 | 12/12  (4.02±0.41) | 12/12 |

Supplementary Table 1. Comparative analysis of *Salmonella* Typhimurium positive tissue samples between direct plating and post enrichment *Salmonella* Typhimurium load (Log10CFU/g) in organ at the time of cull.

Log_10_ CFU data are expressed as mean ± S.E.M.
